# PILOT STUDY TO PRAGMATICALLY EMBED CAREGIVER DEMENTIA EDUCATION AND SUPPORT IN HEALTH CARE SYSTEMS

**DOI:** 10.1093/geroni/igac059.1282

**Published:** 2022-12-20

**Authors:** Richard Fortinsky

**Affiliations:** University of Connecticut Center on Aging, Farmington, Connecticut, United States

## Abstract

Evidence-based interventions offering meaningful benefits to informal caregivers of people living with dementia (PLWD) would be attractive to office-based practitioners if pragmatic linkages could be made between these interventions and outpatient care settings. This presentation will explain experiences and lessons learned in an ongoing pilot study in which we are: pragmatically identifying and inviting caregivers of PLWD to join online dementia education and support programs; collecting and storing caregiver outcomes data into electronic health records where these data are accessible to clinicians. Participating outpatient health care settings are a geriatrics practice at UConn Health and a memory care clinic at Emory Healthcare. Caregivers recruited at both sites participate in either Tele-Savvy or Caregiving During Crisis programs. Outcomes data will inform effects of program participation on caregivers’ competence and stress, and help clinicians gain insights into caregivers’ capacity to manage PLWD. Implementation evaluation strategies and results also will be discussed.

